# Comparison of water availability effect on ammonia-oxidizing bacteria and archaea in microcosms of a Chilean semiarid soil

**DOI:** 10.3389/fmicb.2012.00282

**Published:** 2012-08-08

**Authors:** Mauricio Bustamante, Valentina Verdejo, Catalina Zúñiga, Fernanda Espinosa, Julieta Orlando, Margarita Carú

**Affiliations:** Departamento de Ciencias Ecológicas, Facultad de Ciencias, Universidad de ChileSantiago, Chile

**Keywords:** ammonia-oxidizing archaea, ammonia-oxidizing bacteria, water pulses, semiarid soil, microcosms

## Abstract

Water availability is the main limiting factor in arid soils; however, few studies have examined the effects of drying and rewetting on nitrifiers from these environments. The effect of water availability on the diversity of ammonia-oxidizing bacteria (AOB) and archaea (AOA) from a semiarid soil of the Chilean sclerophyllous matorral was determined by microcosm assays. The addition of water every 14 days to reach 60% of the WHC significantly increased nitrate content in rewetted soil microcosms (*p* < 0.001). This stimulation of net nitrification by water addition was inhibited by acetylene addition at 100 Pa. The composition of AOA and AOB assemblages from the soils microcosms was determined by clone sequencing of *amo*A genes (A-*amo*A and B-*amo*A, respectively), and the 16S rRNA genes specific for β-proteobacteria (beta-*amo*). Sequencing of beta-*amo* genes has revealed representatives of *Nitrosomonas* and *Nitrosospira* while B-*amo*A clones consisted only of *Nitrosospira* sequences. Furthermore, all clones from the archaeal *amo*A gene library (A-*amo*A) were related to “mesophilic Crenarchaeota” sequences (actually, reclassified as the phylum Thaumarchaeota). The effect of water availability on both microbial assemblages structure was determined by T-RFLP profiles using the genetic markers *amo*A for archaea, and beta-*amo* for bacteria. While AOA showed fluctuations in some T-RFs, AOB structure remained unchanged by water pulses. The relative abundance of AOA and AOB was estimated by the Most Probable Number coupled to Polymerase Chain Reaction (MPN-PCR) assay. AOB was the predominant guild in this soil and higher soil water content did not affect their abundance, in contrast to AOA, which slightly increased under these conditions. Therefore, these results suggest that water addition to these semiarid soil microcosms could favor archaeal contribution to ammonium oxidation.

## Introduction

Cycles of drought-rewetting are common in most terrestrial ecosystems, but they are particularly pronounced in arid and semiarid environments mainly with seasonal rainfall (Fierer and Schimel, 2002). The Chilean matorral is a semiarid ecosystem located between the coastal and the Andes mountain ranges, between 32 and 36° south latitude with a temperate Mediterranean climate characterized by dry summers and rainy winters. The matorral is predominantly composed by shrub vegetation, which has been affected by anthropogenic activities resulting in an extensive loss of native vegetation and soil erosion with low fertility and limited nitrogen (Fuentes, 1990). In the Andean foothills some fragments of matorral are composed mainly by *Colletia hystrix*. These shrubs are considered pioneer species that contribute to primary plant successions stabilizing the soil and increasing its nitrogen content (Silvester et al., 1985) because of their ability to form root nodules with *Frankia*, a N_2_-fixing actinomycete (Carú, 1993; Carú et al., 2003). Moreover, this semiarid soil sustained denitrification activity and showed a broad genetic variability of denitrifiers (Orlando et al., 2012). Other major players in the nitrogen cycle are nitrifiers, archaea and/or bacteria, which have not been studied in this particular environment and have generally been addressed in some arid environments only.

In semiarid ecosystems, water availability plays a critical role in the soil affecting the osmotic status, abundance, and composition of microbial cells as well as influencing nutrient cycling (Fierer et al., 2003; Griffiths et al., 2003; Drenovsky et al., 2004; Singh and Kashyap, 2006; Schimel et al., 2007). However, nitrogen is often the most limiting nutrient (Whitford, 1992), and rewetting of dried soils produces an increment in its mineralization (Lundquist et al., 1999). As ammonium oxidation is the limiting step of nitrification and plays a central role in the global nitrogen cycle, the ammonia-oxidizing bacteria (AOB) have been used as indicators of specific environmental conditions in microbial ecology studies (Kowalchuk and Stephen, 2001). Their detection has been mainly performed using the 16S rRNA and *amo*A (ammonia monooxygenase subunit A) genes specific to AOB. They are affiliated to the beta- and gamma-subdivision of the Proteobacteria (Purkhold et al., 2000). However, to date, gammaproteobacterial AOB have been observed only in marine environments (Kowalchuk and Stephen, 2001; Ward and O'Mullan, 2002).

Until recently, AOB have been considered to be responsible for the majority of ammonium oxidation in soil. However, the recent discovery of the gene responsible for ammonium oxidation (*amo*A) in the domain archaea, and the detection of putative ammonia-oxidizing archaea (AOA) in different ecosystems, suggests that AOA may play a significant, but previously unrecognized role in the global nitrogen cycle (Francis et al., 2005; Leininger et al., 2006; He et al., 2007; Prosser and Nicol, 2008). These archaea possess genes homologs to those encoding subunits A, B, and C (*amo*A, *amo*B, and *amo*C homologs) of the ammonia monooxygenase (AMO) of autotrophic AOB. During the last few years, crenarchaeota possessing putative *amo*A genes have been identified in marine and terrestrial environments (Treusch et al., 2005; Leininger et al., 2006), although recently, the AOA have been assigned to the new archaeal phylum Thaumarchaeota (Pester et al., 2011).

Despite the critical importance of nitrification in the nitrogen cycle, our understanding of the role of the different ammonia oxidizers (i.e., AOB and AOA) in different soils and environmental conditions is still limited (Schleper, 2010), mainly because until now there are no selective inhibitors for any of these microbial groups. Nevertheless, there is a growing body of reports addressing questions about AOB and AOA ecological roles today, but it should be considered that to know what factors regulate ammonia-oxidizing populations, AOA and AOB must be studied in a wide variety of habitats. Although ammonia-oxidizers are sensitive to water availability (Stark and Firestone, 1995), especially considering that this is the main limiting factor in arid environments; few studies have examined the effects of drying and rewetting on bacterial nitrification (Stark and Firestone, 1995; Avrahami and Bohannan, 2007; Gleeson et al., 2008). However, to our knowledge, just the work of Gleeson et al. (2010) has determined the direct influence of water availability on AOB and AOA community structures from a semiarid soil, evidencing a clear separation between “wet” and “dry” samples.

The influence of rewetting stress operates at both physiological and community composition levels and these levels interact to link environmental conditions and biogeochemical processes (Schimel et al., 2007). The overall aim of this study has been to evaluate the effect of drying-rewetting treatments, in microcosms assays, on the net nitrification and community structure of AOA and AOB in a semiarid soil from Central Chile. The proposal is that, as water availability is a limiting factor for microbial activity in arid soils, the water addition will produce an increase in nitrification activity. This higher activity could not necessarily be associated with a change in nitrifiers structure due to the long generation times known for some of these microorganisms (Avrahami et al., 2002), and because the applied drying-rewetting treatments were sustained over incubation time although not drastic.

## Materials and methods

### Study site and soil sampling

Semiarid soil samples were obtained from the sclerophyllous matorral in the locality of “El Romeral” (33°48′S, 70°14′W), Santiago, Chile. The area presents a dry Mediterranean climate with winter rainfall averaging 350 mm annually and corresponds to vegetation fragments located in the Andean foothills. Ten random sampling points separated from each other between 1 and 2 m within a plot of 15 m × 15 m were selected, soil samples were collected from the upper 10 cm with 6 cm diameter corers and sieved to 2 mm aggregate size. The samples were homogenized to obtain a composite sample to reduce the spatial heterogeneity at a small scale (Webster et al., 2002) and stored at 4°C, for approximately one week, until the microcosms set-up.

### Microcosms design and sampling schedule

The microcosms were set-up with 150 g of the soil samples in clean plastic containers in a 1:3 (v:v) soil:air ratio, and kept at 20°C during 56 days. The treatments consisted in the addition of sterile water and were applied in triplicate to reach 60% of the water-holding capacity (WHC) at the beginning of the experiment and every 14 days (tH2O). The control microcosms were not wetted (w/t). In addition, one set of rewetted microcosms was treated with acetylene (100 Pa) to inhibit the autotrophic ammonium oxidation (tH2O-Ac). To maintain aerobic conditions in the microcosms, they were opened and vented every 3 days and acetylene partial pressures were re-established each time. Since in control microcosms without addition of water a notorious increase in nitrate content during a pre-incubation was not observed (data not shown), the addition of acetylene was performed only in the wetted microcosms.

Samples were obtained from each microcosm set every 7 days to determine pH, moisture content (MC), organic matter content (OM), and the ammonium and nitrate concentrations. Samples coinciding with re-wetting were taken 3 h after treatment application. Clone libraries were obtained at the beginning of the incubation (t0), while T-RFLP profiles were determined at 0, 28, and 56 days of incubation. Finally, samples to abundance calculations were taken at the beginning and at the end of the incubation.

### Edaphic factors

The pH was measured from a soil extract in 2 M KCl using potentiometry (pH 500 Benchtop Meter, Oakton® Instruments). The MC and the OM were calculated gravimetrically before and after desiccation and calcination, respectively (Forster, 1995). Ammonium and nitrate concentrations were determined by colorimetric methods from a soil extract in 2M KCl and deionized water, respectively (Nelson, 1983; Yang et al., 1998).

### DNA extraction and PCR conditions

DNA from each microcosm was obtained from 0.25 g of soil sample using the Ultra Clean Soil DNA kit (MoBio Lab, Inc.) according to the manufacturer's instructions. PCR reactions contained 10–20 ng template DNA, GoTaq® Green Master Mix (GoTaq® DNA Polymerase in 1x Green GoTaq® Reaction Buffer pH 8.5, 200 μM of each dNTP and 1.5 mM MgCl_2_) (Promega®) and each primer at the appropriate concentration.

The PCR amplification of the 16S rRNA gene specific for beta-proteobacterias (beta-*amo*) was carried out by a nested PCR strategy. First, 200 nM of the universal primers fD1 (5′-AGAGTTTGATCCTGGCTCAG-3′) and rP2 (5′-ACGGCTACCTTGTTACGACTT-3′) (Weisburg et al., 1991) were used. The second PCR was assessed using 200 nM of primers βAMOf (5′-TGGGGRATAACGCAYCGAAAG-3′) and βAMOr (5′-AGACTCCGATCCGGACTACG-3′) (McCaig et al., 1994).

The *amo*A gene of AOB (B-*amo*A) was amplified also using a nested PCR (Yeager et al., 2005). In the first PCR, the forward primer amoA-2F (5′-AARGCGGCSAAGATGCCGCC-3′) and the reverse primer amoA-5R (5′-TTATTTGATCCCCTC-3′) (Webster et al., 2002) each at 230 nM were used. For the second PCR, forward primer amoA-1F (5′-GGGGTTTCTACTGGTGGT-3′) and reverse primer amoA-2R (5′-CCCCTCKGSAAAGCCTTCTTC-3′) (Rotthauwe et al., 1997) were used each at 660 nM.

The *amo*A gene of AOA (A-*amo*A) was amplified using primers Crenamo23f (5′-ATGGTCTGGCTWAGACG-3′) and Crenamo616r (5′-GCCATCCATCTGTATGTCCA-3′) (Tourna et al., 2008), each at 200 nM. For T-RFLP analysis the forward primers βAMOf and Crenamo23f 5′end-labeled with FAM were used.

### Clone libraries

The PCR-products were purified using the Wizard® DNA Clean-Up System (Promega®). The cleaned amplicons were ligated to the vector pTZ57R/T and used to transform *Escherichia coli* XL1B by means of the InsTAclone™ PCR Cloning Kit (Fermentas®) according to manufacturer's guidelines. Transformants were grown in LB medium (1.0% Tryptone; 0.5% yeast extract; 1.0% NaCl; pH 7.0) and selected through their resistance to ampicillin (50 mg ml^−1^) and their inability to hydrolyze X-gal in presence of IPTG. The presence of the expected insert size was confirmed by PCR using the vector's primers M13F and M13R. As template a cells suspension in buffer TE subject to eight successive cycles of thermal shock 1 min to 98°C/1 minute to 4°C was used. PCR products of *amo*A and beta-*amo* genes were digested using 20U of *Hha*I and *Hae*III (Promega®) restriction enzymes, respectively, and a representative clone of each profile was sequenced (Macrogen Inc.) in one direction using a Genetic Analyzer 3730XL (Applied Biosystems). The clone sequences identity was evaluated using the BLASTN program (Altschul et al., 1997). The sequenced clones were stored in 20% glycerol at −80°C.

### Terminal restriction fragment length polymorphism (T-RFLP) profiles

The beta-*amo* and the A-*amo*A amplicons were purified using the Wizard® DNA Clean-Up System (Promega®) according to the manufacturer's instructions. The purified PCR products (100 ng) of beta-*amo* were digested in separate reactions with 20 U of *Hha*I and *Hae*III (Promega®) restriction enzymes, while 20 U of *Hha*I and *Mnl*I (Promega®) were used to digest A-*amo*A products (100 ng). The terminal restriction fragments (T-RFs) were separated with an automated Genetic Analyzer 3100 (Applied Biosystems, Macrogen Inc.). The lengths of fluorescently labeled T-RFs were determined by the comparison with the internal standard GeneScan-500™ LIZ® using the GeneScan 3.71 software (Applied Biosystems).

The peaks with a fluorescence of 30 U and larger than 30 bp were analyzed by peak height. Patterns from different samples were normalized to identical total fluorescence units by an iterative standardization procedure (Dunbar et al., 2001). This normalization procedure is necessary because of the inherent variability in total DNA quantity among samples on the sequencing gel. Relative abundance of T-RFs, as a percentage, was determined by calculating the ratio between height of a given peak and the normalized total peak height of each sample. Manual alignment of T-RFs profiles was necessary because the Genescan software calculates DNA fragment sizes to 1/100 of a base pair, but the error associated with fragment analysis can be up to 0.5 bp. T-RFs of different lengths were assumed to represent distinct operational taxonomic units (OTUs), although not necessarily unique species.

### Most probable number—polymerase chain reaction (MPN-PCR) assay

To estimate the abundance of AOA and AOB, as gene units (GU), the MPN-PCR was used. Ten-fold dilution series, in triplicate, of the extracted DNA from w/t and tH2O microcosms at the beginning and at the end of incubation times were performed. Then, 1 μl was used as template in a 25-μl PCR mixture to amplified B-*amoA* and A-*amo*A genes as aforementioned; the PCR products visualized on 1.2% agarose gels staining with ethidium bromide (0.5 mg l^−1^) were used as the positive signal. MPN values were estimated according to Jarvis et al. (2010) based on a MS-Excel spreadsheet freely available from http://www.wiwiss.fuberlin.de/institute/iso/mitarbeiter/wilrich/index.html.

### Statistical analysis

Edaphic factors and T-RFs data from triplicate microcosms of each treatment were analyzed by multiple comparisons through Two-Way analysis of variance (Two-Way ANOVA) followed by Bonferroni's post-test by the GraphPad Prism v4.0 program (GraphPad Software, Inc.). MPN lower and upper confidence limits were estimated according to Jarvis et al. (2010).

## Results

This study was designed to determine the effect of drying-rewetting in a semiarid soil on nitrification activity and nitrifiers structure. The experimental approach consisted of monitoring a 14-days rewetting regime during 56 days of soil microcosms incubation. Edaphic factors such as pH (6.3–7.0) and OM (5.7–6.7%) did not vary with respect to the treatment according to Bonferroni's test (*p* < 0.05). On the other hand, the MC reflected the treatment effect, with significantly different values for wetted (10.9–15.7%) and non-treated microcosms (2.3–4.4%) (Bonferroni's test, *p* < 0.01) (Table 1).

**Table 1:** **Edaphic factors during microcosms incubation time (mean values; numbers in parenthesis are standard deviation)**.

**Treatment^a^**		**pH**			**OM (%)**			**MC (%)**		
		**w/t**	**tH2O**	**tH2O-Ac**	**w/t**	**tH2O**	**tH2O-Ac**	**w/t**	**tH2O**	**tH2O-Ac**
Incubation time^b^	0	6.51 (0.08)a	6.41 (0.12)a	6.29 (0.10)a	6.01 (0.53)a	6.04 (0.28)a	6.02 (0.06)a	4.39 (0.05)a	15.01 (0.09)b	15.04 (0.09)b
	7	6.61 (0.14)a	6.57 (0.12)a	6.64 (0.02)a	6.22 (0.34)a	6.23 (0.31)a	6.08 (0.03)a	3.12 (0.04)a	10.86 (0.89)b	10.99 (0.06)b
	14	6.44 (0.14)a	6.36 (0.05)a	6.40 (0.14)a	5.86 (0.22)a	6.08 (0.17)a	5.98 (0.12)a	2.38 (0.20)a	14.72 (0.33)b	15.12 (0.08)b
	21	6.58 (0.41)a	6.43 (0.23)a	6.51 (0.19)a	6.15 (0.35)a	5.91 (0.08)a	5.82 (0.08)a	2.53 (0.11)a	11.11 (0.12)b	11.05 (0.01)b
	28	6.43 (0.11)a	6.49 (0.03)a	6.60 (0.03)a	6.73 (0.65)a	6.06 (0.20)b	5.84 (0.08)b	2.29 (0.01)a	15.73 (0.41)b	15.62 (0.04)b
	35	6.46 (0.17)a	6.48 (0.18)a	6.55 (0.06)a	5.72 (0.71)a	5.80 (0.11)a	6.05 (0.05)a	2.40 (0.22)a	11.02 (1.04)b	11.75 (0.05)b
	42	6.77 (0.04)a	6.72 (0.13)a	6.55 (0.20)a	5.69 (0.24)a	5.68 (0.03)a	5.75 (0.10)a	2.34 (0.11)a	15.29 (0.51)b	15.49 (0.09)b
	49	6.93 (0.07)a	6.71 (0.08)a	6.73 (0.08)a	5.97 (0.14)a	6.08 (0.25)a	6.04 (0.18)a	2.30 (0.08)a	11.18 (1.47)b	11.26 (0.18)b
	56	6.97 (0.14)a	6.64 (0.19)a	6.77 (0.16)a	5.66 (0.02)a	5.81 (0.03)a	5.76 (0.07)a	2.54 (0.08)a	15.57 (0.19)b	15.74 (0.09)b

a Treatment: w/t, microcosms without treatment; tH2O, microcosms treated with water each 14 days; tH2O-Ac, microcosms treated with water each 14 days and with 100 Pa acetylene.

b Incubation time is indicated in days. For each edaphic factor, mean values within rows followed by the same letter are not significantly different (Bonferroni's test, p < 0.01).

**Figure 1:**
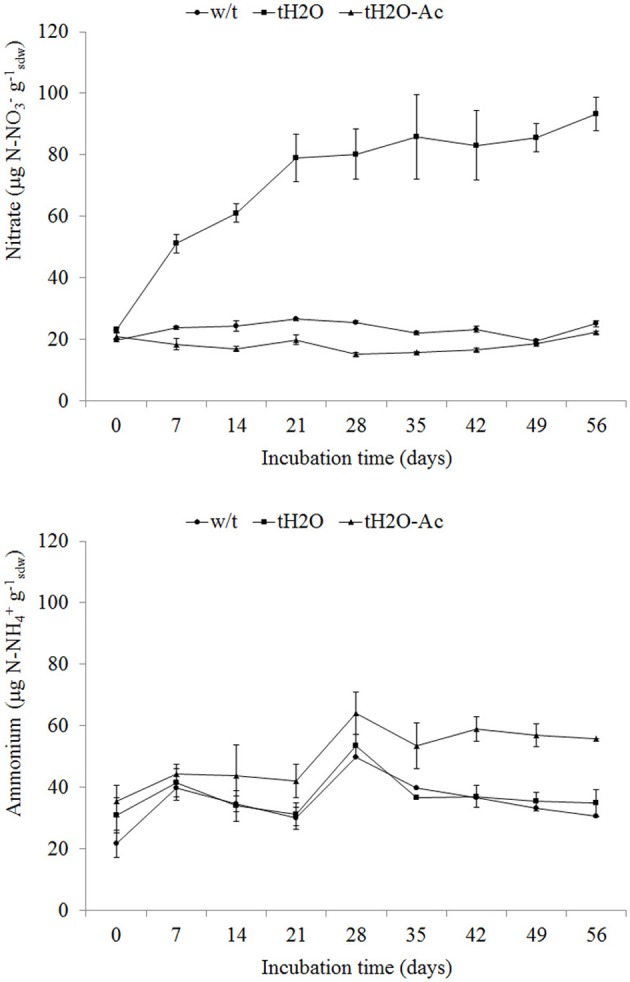
**Nitrate (upper panel) and ammonium (lower panel) concentrations (mean values; error bars represent standard deviation) in microcosms without treatment (w/t; circle), microcosms treated with water each 14 days (tH2O; square) and microcosms treated with water each 14 days and 100 Pa acetylene (tH2O-Ac; triangle) during the incubation time.** Nitrate concentration data of tH2O microcosms differ from those of w/t and tH2O-Ac microcosms from incubation time of 7 days (*p* < 0.001; Bonferroni test). Ammonium concentration data of tH2O-Ac microcosms differ from those of w/t and tH2O microcosms from incubation time of 21 days (*p* < 0.05; Bonferroni's test).

Net nitrification occurred in rewetted microcosms (tH2O) throughout the incubation period (net nitrification rate: 1.04 μg N-NO_3_ g^−1^_sdw_ d^−1^), in which the nitrate content was above five times higher than in the non-treated samples (w/t) (*p* < 0.001) (Figure 1). This nitrate accumulation should be the result of nitrate producing and consuming processes. Moreover, this nitrate content increase was inhibited when the wetted soil was incubated in the presence of acetylene (tH2O-Ac) (Figure 1). Acetylene is a potent inhibitor of autotrophic nitrification (Hynes and Knowles, 1982) because the oxidation of acetylene by AMO results in permanent inhibition of the enzyme (Hyman and Wood, 1985). Recently, Offre et al. (2009) also showed that in microcosms containing acetylene, the nitrification of archaeal phylotypes was suppressed. On the other hand, significantly differences (*p* < 0.05) in ammonium concentration were observed between wetted (tH2O) and control samples (w/t) with respect to wetted microcosms treated with acetylene (tH2O-Ac) (Figure 1). As no ammonium was added to the microcosms, the ammonium is probably continuously regenerated by nitrogen mineralization in a water-dependent process.

In the next step, the wetted treatment effect on the structure of AOB and/or AOA was assessed. First, the soil AOB composition was determined using two different molecular markers: the bacterial *amo*A gene (B-*amo*A) and the beta-proteobacterial 16S rRNA gene (beta-*amo*). All B-*amo*A sequences were only related to bacteria of the genus *Nitrosospira* (Table 2), while clones beta-*amo*, despite including non-AOB (27.8% of Pseudomonadaceae, 19.4% of Oxalobacteraceae and 8.3% of Comamonadaceae), detected the presence of the two most important groups of AOB (22.2% of *Nitrosospira* sp. and 5.6% of *Nitrosomonas* sp.) (Table 2). For this reason, to evaluate the effect of rewetting on the structure of AOB and AOA communities, T-RFLP analysis was assessed using the beta-*amo* and the A-*amo*A genes, respectively. The structure of nitrifiers communities was determined from relative abundances of dominant T-RFs. Bacterial and archaeal T-RFLP profiles from wetted and non-treated microcosms obtained with different restriction enzymes, showed similar results with water addition, therefore, only the profiles obtained by digestion with *Hha*I are showed.

**Table 2:** **Relative frequency of haplotypes in the AOA and AOB gene libraries**.

**Group**	**RFLP profile^a^ (Haplotype)**	**Frequency^b^**	**Most related sequence (accession number)^c^**
AOA (A-*amo*A gene)	A (A-*amo*A1)	59.2	Crenarchaeote clone DZ3_65 (JF748225.1)
	B (A-*amo*A4)	31.0	Crenarchaeote clone 11 (DQ304872.1)
	C (A-*amo*A2)	4.2	Crenarchaeote clone AOA-B12 (HM113513.1)
	D (A-*amo*A6)	1.4	Archaeon clone ES-Core-SR1-sH04 (HM363947.1)
	E (A-*amo*A9)	1.4	Crenarchaeote clone DL3_54 (JF748179.1)
	F (A-*amo*A10)	1.4	Crenarchaeote clone LZT1-A17 (GQ226122.1)
	G (A-*amo*A11)	1.4	Crenarchaeote clone DL2_48 (JF748152.1)
AOB (B-*amo*A gene)	A (B-*amo*A1)	58.3	*Nitrosospira sp*. clone Nt1 (AY445617.1)
	B (B-*amo*A35)	5.0	*Nitrosospira sp.* AHB1 (X90821.1)
	C (B-*amo*A38)	20.0	*Nitrosospira sp*. EnI299 (EF175100.1)
	D (B-*amo*A10)	3.3	*Nitrosospira sp.* LT1FMf (AY189144.1)
	E (B-*amo*A7)	3.3	*Nitrosospira sp.* LT2MFa (AY189145.1)
	F (B-*amo*A5)	1.7	*Nitrosospira sp*. EnI299 (EF175100.1)
	G (B-*amo*A9)	1.7	*Nitrosospira sp.* LT2MFa (AY189145.1)
	H(B-*amo*A15)	1.7	*Nitrosospira sp*. LT2MFa (AY189145.1)
	I (B-*amo*A22)	1.7	*Nitrosospira sp.* clone 1-61 (GU136445.1)
	J (B-*amo*A37)	1.7	*Nitrosospira sp*. CT2F (AY189143.1)
	K (B-*amo*A43)	1.7	*Nitrosospira sp*. EnI299 (EF175100.1)
AOB (beta-*amo* gene)	A (Beta-*amo*26)	8.3	*Nitrosospira sp.* EnI299 (EF175101.1)
	B (Beta-*amo*19)	5.6	*Nitrosospira sp.* Nsp57 (AY123791.1)
	C (Beta-*amo*5)	2.8	*Nitrosospira sp.* L115 (AY123796.1)
	D (Beta-*amo*7)	2.8	*Nitrosomonas sp.* Nm86 (AY123798.1)
	E (Beta-*amo*11)	2.8	*Nitrosospira sp*. Nsp57 (AY123791.1)
	F (Beta-*amo*17)	2.8	*Nitrosomonas sp.* Nm86 (AY123798.1)
	G (Beta-*amo*18)	2.8	*Nitrosospira sp.* Nsp65 (AY123813.1)
	Others	72.2	Non-AOB

a The RFLP profiles were obtained with HhaI and HaeIII restriction enzymes for amoA and beta-amo genes, respectively.

b The frequency was calculated as relative percentage of total clones.

c The most related sequence detected by sequence alignment with the NCBI database is shown. Query coverage and maximal identity were over 90% in all cases.

**Figure 2:**
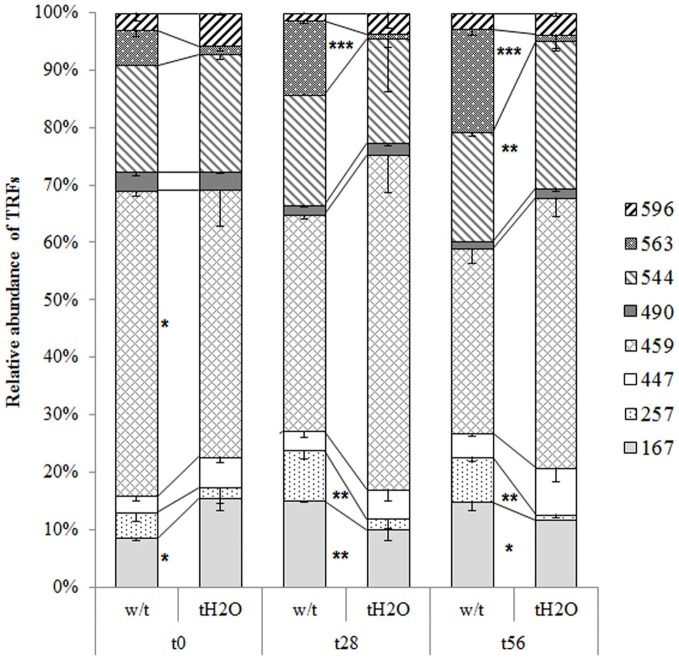
**Relative abundances (mean values; error bars represent standard deviation) of the AOA *amo*A genes T-RFs from microcosms without treatment (w/t) and microcosms treated with water each 14 days (tH2O) during the incubation time.** The different textures represent different T-RFs in base pairs (bp). At each incubation time, relative abundances of T-RFs significantly differents are indicated as follows: ^*^p = 0.05; ^**^p = 0.01; ^***^p = 0.001 (Bonferroni's test).

The T-RFLP profiles revealed that the AOA community was significantly affected by treatment. In both microcosms the T-RFs richness was the same at each incubation time, and no significant abundance change was observed for T-RFs of 447, 490, and 596 bp (Figure 2). Among the most significant changes in abundance due to water treatment, are the decline in abundance of T-RFs of 257 and 563 bp from day 28 of incubation, and the increased abundance of the T-RF of 544 bp at the end of incubation. Most T-RFs could not be assigned by *in silico* restriction to an AOA clone, with the exception of the T-RF of 167 bp which corresponds to the most abundant haplotype A (Table 2). All of haplotypes were related with sequences obtained from soil samples but not with clones derived from extreme environments or marine samples. Summarizing, the AOA structure was affected by the water addition, which could be related to the increase of nitrification activity observed in the treated microcosms.

**Figure 3:**
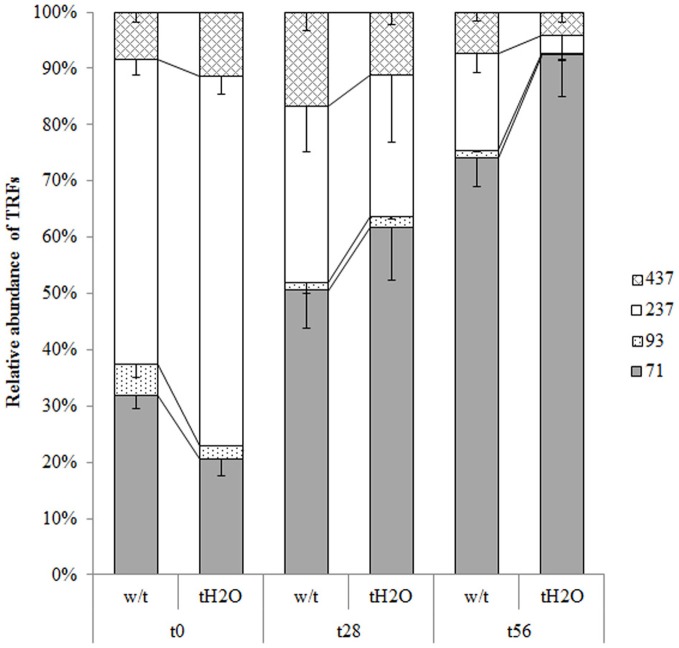
**Relative abundances (mean values; error bars represent standard deviation) of the AOB beta-*amo* genes T-RFs from microcosms without treatment (w/t) and microcosms treated with water each 14 days (tH2O) during the incubation time.**The different textures represent different T-RFs in base pairs (bp). At each incubation time, relative abundances of T-RFs are not significantly different (Bonferroni's test, *p* < 0.05).

On the other hand, changes in T-RFLP patterns of AOB between control and treated microcosms could not be observed, indicating that the beta-proteobacterial ammonia-oxidizers community structure remained unchanged under the applied treatment (Figure 3). However, in this case, changes were detected throughout the incubation time when comparing both microcosms; the most notable changes being the increasing T-RF of 71 bp and the decreasing T-RF of 237 bp from day 28 of incubation (Figure 3). The peaks of this profile were identified using the simulation tool MICA (http://mica.ibest.uidaho.edu/) and only two of them could be attributed to ammonia-oxidizers bacteria (T-RF of 437 bp to *Nitrosospira* sp. and T-RF of 237 bp to *Nitrosomonas* sp.). However, when the beta-*amo* genes sequences were digested with *Hha*I *in silico*, the T-RF of 71 bp was identified as belonging to *Nitrosospira* sp., and the association of the T-RF of 237 bp to *Nitrosomonas* sp. was confirmed, although other non-AOB clones could also produce the same T-RFs.

Finally, assays were carried out to determine whether the increase of nitrate content in the rewetted soil microcosms was linked to an increase in the number of AOA or AOB. To determine the abundance of AOA and AOB, the MPN-PCR method was used. In this case, the B-*amo*A molecular marker was used for AOB because of the impossibility of distinguishing the contribution of non-AOB to the gene pool if the beta-*amo* is used. Therefore, prior to the determination of abundance, the proportion of each haplotype in both groups (AOA and AOB) was calculated (Table 2). All haplotypes of AOA were related to Crenarchaeota clones, the most abundant one (59.2%) was related to an upland red soil under long-term fertilization practices (He et al., 2007). On the other hand, all AOB clones were related to bacteria of the genus *Nitrosospira*; moreover, several haplotypes were more closely related to sequences from isolated strains. However, the most represented haplotype (58.3%) was associated with a clone from an agriculture soil (Okano et al., 2004).

The MPN-PCR method indicated that the AOB abundances were higher than AOA abundances in both microcosms and incubation times assessed (Table 3). Further, the wetted microcosms (tH2O) showed a significantly higher number of AOA at the end of incubation compared to non-treated microcosms (w/t). However, the abundance of AOB increased with incubation time regardless of treatment and at the end of incubation the number of bacteria was higher in microcosms without treatment (w/t) (Table 3).

**Table 3:** **Abundances of AOA and AOB in the microcosms as determined by MPN-PCR of the corresponding *amo*A gene (mean values expressed as MPN (UG g^−1^_dws_); numbers in parenthesis are lower and upper confidence limits)**.

	**t0**	**t56 (w/t)*^a^***	**t56 (tH2O)*^b^***
AOA	2.4 × 10^3^ (5.6 × 10^2^–1.0 × 10^4^)	2.4 × 10^3^ (5.6 × 10^2^–1.0 × 10^4^)	1.3 × 10^4^ (3.1 × 10^3^–5.5 × 10^4^)
AOB	2.8 × 10^5^ (6.4 × 10^4^–1.2 × 10^6^)	1.5 × 10^8^ (4.3 × 10^7^–5.0 × 10^8^)	1.6 × 10^7^ (5.3 × 10^6^–5.0 × 10^7^)

a w/t, microcosms without treatment and incubated during 56 days.

b tH2O, microcosms treated with water each 14 days and incubated during 56 days.

## Discussion

The aim of this study was to evaluate the effect of periodic rewetting of semiarid soil microcosms from the Chilean sclerophyllous matorral on nitrification and on AOB and AOA communities structure. Episodic water availability affects element cycling in arid and semiarid ecosystems (Schimel et al., 1997; Gebauer and Ehleringer, 2000; Schaeffer and Evans, 2005; Singh and Kashyap, 2006); and many small rain events, as simulated in this study, can cause a rapid increase in soil microorganisms activity (Schwinning and Sala, 2004). Moreover, the increased net nitrification observed in the wetted microcosms (tH2O) agrees with other studies where a positive effect of soil moisture increase on the microbial activity was observed (Stark and Firestone, 1995; Avrahami and Bohannan, 2007; Gleeson et al., 2008, 2010). Additionally, the low nitrate content in the microcosm treated with acetylene supports nitrification observed in the wetted microcosm. Inhibition of soil ammonium oxidation by acetylene has been investigated extensively (McCarty and Bremner, 1986; Garrido et al., 2000) and has been demonstrated for both AOB (Hyman and Wood, 1985) and AOA (Offre et al., 2009). However, it should be noted that net changes in the nitrate pool do not solely reflect nitrifying activity, but it includes the consumption process as the activity carried out by denitrifiers described in this soil (Orlando et al., 2012). Directly linked to the observed nitrification activity, a decrease of ammonium concentrations was observed in the wetted microcosms (tH2O). When acetylene was added to wetted microcosms (tH2O-Ac) to inhibit nitrification activity, ammonium content was higher than in the control microcosms (w/t). As it was not added to the microcosms, the ammonium could be continuously generated by nitrogen mineralization since the frequent soil moisture fluctuations result in pulses of nitrogen mineralization after wetted events as have been described in various reports (Fierer and Schimel, 2002; Austin et al., 2004).

On the other hand, shifts by rewetting of soil in AOA community structure but not in AOB community structure were detected based on T-RFLP profiles. These results agree with those obtained by Offre et al. (2009) which showed that DGGE fingerprint of archaeal *amo*A gene changed, in the relative abundance of specific archaeal phylotypes, in agricultural soil microcosms with active nitrification incubated during 30 days. In contrast, Jia and Conrad (2009) found no changes in the molecular fingerprint of archaeal *amo*A gene in fertilized soil microcosms in which ammonium oxidation was stimulated; moreover, the activity changes were correlated with changes in abundance of bacterial *amoA* gene. In a more recent work, Gleeson et al. (2010) showed that AOA and AOB structures were both affected by changing water filled pore space (WFPS), with the exception of bacterial *amo*A structure assessed at 25% and 55% WFPS. Previously, Gleeson et al. (2008) showed that water availability affects the AOB structure, and changes in potential nitrification activity were significantly correlated to changes in the structure of nitrifying bacterial communities. In our study, significantly differences in bacterial T-RFLP patterns between control and treated microcosms were not observed, although changes in the relative abundance were detected throughout the incubation time. However, it should be considered that the beta-*amo* bacterial T-RFs represent not only AOB, but other non-AOB genera; therefore these could be responsible for changes in T-RFLP patterns during incubation. In addition, other studies have suggested that frequent drying and rewetting may select for fast growing microorganisms that are capable of rapid growth on the labile substrates released into the soil during a rewetting event (Lundquist et al., 1999; Denef et al., 2001).

Finally, nitrifiers abundances determined by MPN-PCR have indicated that in this soil AOB GUs per gram were higher than those related to AOA. This prevalence of AOB was also determined by Gleeson et al. (2010) in a semiarid soil of Western Australia, which represents another of the five Mediterranean type environments existing. In contrast, several reports have shown that soil AOA are more abundant than their bacterial counterpart (Leininger et al., 2006; He et al., 2007; Nicol et al., 2008; Shen et al., 2008; Tourna et al., 2008; Offre et al., 2009; Zhang et al., 2010), including another semiarid soils (Adair and Schwartz, 2008); moreover, several reports indicate that the ammonium oxidation is mostly due to the AOA group (Leininger et al., 2006; He et al., 2007; Nicol et al., 2008; Tourna et al., 2008; Offre et al., 2009). In this study, AOB abundance was determined using bacterial *amo*A gene, and all B-*amo*A sequences from the clone library were only related to *Nitrosospira*, which has been detected as the dominant ammonia-oxidizer group in different neutral pH soils (Kowalchuk et al., 2000; Kowalchuk and Stephen, 2001) suggesting that AOB were the main group responsible for ammonium oxidation in some soil types (Jia and Conrad, 2009). This dominance of AOB over AOA could be explained by adaptation to different soil nitrogen conditions, i.e., AOB would be favored by high ammonium concentrations while AOA grow better at very low ammonium concentrations (Di et al., 2010; Pester et al., 2011). The relatively high concentration of ammonium (20–40 μg N-NH^+^_4_ per gram of dry soil) detected in the wetted and untreated microcosms of this semiarid soil during the incubation time could explain the higher abundances of AOB compared with AOA. Furthermore, at the end of incubation of both microcosms differences neither in the ammonium content nor in the number of AOB were observed. The source of ammonium may differentially influence AOA and AOB as it was shown that AOA would be favored by ammonium derived from soil OM or in environments with low ammonium concentrations (Stopnišek et al., 2010; Pester et al., 2011; Levičnik-Höfferle et al., 2012) while AOB abundance was shown to increase when soils were amended by mineral nitrogen (Jia and Conrad, 2009). In this work a significant increase in AOB abundance was observed despite rewetting regime, instead AOA increased only slightly in water-treated microcosms at the end of incubation of semiarid soils with low OM but no depletion of ammonium. Likewise, Gleeson et al. (2010) showed that abundance of AOA displayed little response to changes in WFPS availability, although a relationship between water availability and the abundance of bacterial but not archaeal nitrifiers was detected.

In conclusion, the addition of water to these soil microcosms increases the nitrate and ammonium concentration. Results suggest that ammonium was produced through mineralization and was accumulated when nitrification was inhibited by acetylene. Despite water-treatment, communities of AOB had similar structures and abundances, indicating that AOB had little contribution to the nitrification process due to the rewetting regime assessed.

Moreover, the ammonium concentration in dry and wetted soil microcosms could be sufficient to sustain a water-independent nitrification that leads to an increased abundance of AOB at the end of the incubation time. By contrast, changes in structure and abundance of AOA communities in the wetted microcosms suggest that most of the oxidation of the mineralized ammonium, upon water addition, could be carried out by archaea. However, the role of AOA and AOB, their contribution to the nitrification process (Offre et al., 2009) and the environmental factors that define the ecological niche of each group (Tuba et al., 2009; Schleper, 2010) are under discussion at present.

### Conflict of interest statement

The authors declare that the research was conducted in the absence of any commercial or financial relationships that could be construed as a potential conflict of interest.

## References

[B1] Adair K. L. Schwartz E. (2008). Evidence that ammonia-oxidizing archaea are more abundant than ammonia-oxidizing bacteria in semiarid soils of northern Arizona, USA. Microb. Ecol. 56, 420–426 10.1007/s00248-007-9360-9 18204798

[B2] Altschul S. F. Madden T. L. Schäffer A. A. Zhang J. Zhang Z. Miller W. Lipman D. J. (1997). Gapped BLAST y PSI-BLAST: a new generation of protein database search programs. Nucleic Acids Res. 25, 3389–3402 10.1093/nar/25.17.3389 9254694 PMC146917

[B3] Austin A. T. Yahdjian L. Stark J. M. Belnap J. Porporato A. Norton U. Ravetta D. A. Schaeffer S. M. (2004). Water pulses and biogeochemical cycles in arid and semiarid ecosystems. Oecologia 141, 221–235 10.1007/s00442-004-1519-1 14986096

[B4] Avrahami S. Bohannan B. J. M. (2007). Response of *Nitrosospira* sp. strain AF-like ammonia oxidizers to changes in temperature, soil moisture content, and fertilizer concentration. Appl. Environ. Microbiol. 73, 1166–1173 10.1128/AEM.01803-06 17158615 PMC1828661

[B5] Avrahami S. Conrad R. Braker G. (2002). Effect of soil ammonium concentration on N_2_O release and on the community structure of ammonia oxidizers and denitrifiers. Appl. Environ. Microbiol. 68, 5685–5692 10.1128/AEM.68.11.5685-5692.2002 12406765 PMC129938

[B6] Carú M. (1993). Characterization of native *Frankia* strains isolated from Chilean shrubs (Rhamnaceae). Plant Soil 157, 137–145

[B7] Carú M. Mosquera G. Bravo L. Guevara R. Sepúlveda D. Cabello A. (2003). Infectivity and effectivity of *Frankia* strains from the Rhamnaceae family on different actinorhizal plants. Plant Soil 251, 219–225

[B8] Denef K. Six J. Bossuyt H. Frey S. D. Elliott E. T. Merckx R. Paustian K. (2001). Influence of dry–wet cycles on the interrelationship between aggregate, particulate organic matter, and microbial community dynamics. Soil Biol. Biochem. 33, 1599–1611

[B9] Di H. J. Cameron K. C. Shen J. P. Winefield C. S. O'Callaghan M. Bowatte S. He J. Z. (2010). Ammonia oxidizing bacteria and archaea grow under contrasting soil nitrogen conditions. FEMS Microbiol. Ecol. 72, 386–394 10.1111/j.1574-6941.2010.00861.x 20370827

[B10] Drenovsky R. E. Vo D. Graham K. J. Scow K. M. (2004). Soil water content and organic carbon availability are major determinants of soil microbial community composition. Microb. Ecol. 48, 424–430 10.1007/s00248-003-1063-2 15692862

[B11] Dunbar J. Ticknor L. O. Kuske C. R. (2001). Phylogenetic specificity and reproducibility and new method for analysis of terminal restriction fragment profiles of 16S rRNA genes from bacterial communities. Appl. Environ. Microbiol. 67, 190–197 10.1128/AEM.67.1.190-197.2001 11133445 PMC92545

[B12] Fierer N. Schimel J. (2002). Effects of drying–rewetting frequency on soil carbon and nitrogen transformations. Soil Biol. Biochem. 34, 777–787

[B13] Fierer N. Schimel J. P. Holden P. A. (2003). Influence of drying-rewetting frequency on soil bacterial community structure. Microb. Ecol. 45, 63–71 10.1007/s00248-002-1007-2 12469245

[B14] Forster J. C. (1995). Soil Nitrogen, in Methods in Applied Soil Microbiology and Biochemistry, eds Alef K. Nannipieri P. (San Diego, CA: Academic Press Inc.), 79–87

[B15] Francis C. A. Roberts K. J. Beman J. M. Santoro A. E. Oakley B. B. (2005). Ubiquity and diversity of ammonia-oxidizing archaea in water columns and sediments of the ocean. Proc. Natl. Acad. Sci. U.S.A. 102, 14683–14688 10.1073/pnas.0506625102 16186488 PMC1253578

[B16] Fuentes E. R. (1990). Landscape change in Mediterranean-type habitats of Chile: patterns and processes, in Changing Landscapes: An Ecological Perspective, eds Zonneveld I. S. Forman R. T. T. (Berlin, Germany: Springer-Verlag), 165–190

[B17] Garrido F. Hénault C. Gaillard H. Germon J. C. (2000). Inhibitory capacities of acetylene on nitrification in two agricultural soils. Soil Biol. Biochem. 32, 1799–1802

[B18] Gebauer R. L. Ehleringer J. R. (2000). Water and nitrogen uptake patterns following moisture pulses in a cold desert community. Ecology 81, 1415–1424

[B19] Gleeson D. B. Herrmann A. M. Livesley S. J. Murphy D. V. (2008). Influence of water potential on nitrification and structure of nitrifying bacterial communities in semiarid soils. Appl. Soil Ecol. 40, 189–194

[B20] Gleeson D. B. Müller C. Banerjee S. Ma W. Siciliano S. D. Murphy D. V. (2010). Response of ammonia oxidizing archaea and bacteria to changing water filled pore space. Soil Biol. Biochem. 42, 1888–1891

[B20a] Griffiths R. I. Whiteley A. S. O'Donnell A. G. Bailey M. J. (2003). Physiological and community responses of established grassland bacterial populations to water stress. Appl. Environ. Microbiol. 69, 6961–6968 10.1128/AEM.69.12.6961-6968.2003 14660337 PMC309888

[B21] He J. Z. Shen J. P. Zhang L. M. Zhou Y. G. Zheng Y. M. Xu M. G. Di H. (2007). Quantitative analyses of the abundance and composition of ammonia-oxidizing bacteria and ammonia-oxidizing archaea of a Chinese upland red soil under long-term fertilization practices. Environ. Microbiol. 9, 2364–2374 10.1111/j.1462-2920.2007.01358.x 17686032

[B22] Hyman M. R. Wood P. M. (1985). Suicidal inactivation and labelling of ammonia mono-oxygenase by acetylene. Biochem. J. 227, 719–725 4004794 10.1042/bj2270719 PMC1144898

[B23] Hynes R. K. Knowles R. (1982). Effect of acetylene on autotrophic and heterotrophic nitrification. Can. J. Microbiol. 28, 334–340

[B24] Jarvis B. Wilrich C. Wilrich P. T. (2010). Reconsideration of the derivation of Most Probable Numbers, their standard deviations, confidence bounds and rarity values. J. Appl. Microbiol. 109, 1660–1667 10.1111/j.1365-2672.2010.04792.x 20602657

[B25] Jia Z. Conrad R. (2009). Bacteria rather than Archaea dominate microbial ammonia oxidation in an agricultural soil. Environ. Microbiol. 11, 1658–1671 10.1111/j.1462-2920.2009.01891.x 19236445

[B26] Kowalchuk G. A. Stienstra A. W. Heilig G. H. J. Stephen J. R. Woldendorp J. W. (2000). Composition of communities of ammonium-oxidising bacteria in wet, slightly acid grassland soils using 16S rDNA-analysis. FEMS Microbiol. Ecol. 31, 207–215 10.1111/j.1574-6941.2000.tb00685.x 10719201

[B26a] Kowalchuk G. A. Stephen J. R. (2001). Ammonia-oxidizing bacteria: a model for molecular microbial ecology. Annu. Rev. Microbiol. 55, 485–529 10.1146/annurev.micro.55.1.485 11544365

[B27] Leininger S. Urich T. Schloter M. Scwark L. Qi J. Nicol G. W. Prosser J. I. Schuster S. C. Schleper C. (2006). Archaea predominate among ammonia-oxidizing prokaryotes in soils. Nat. Lett. 442, 806–809 10.1038/nature04983 16915287

[B28] Levičnik-Höfferle S. Nicol G. W. Ausec L. Mandić-Mulec I. Prosser J. I. (2012). Stimulation of thaumarchaeal ammonia oxidation by ammonia derived from organic nitrogen but not added inorganic nitrogen. FEMS Microbiol. Ecol. 80, 114–123 10.1111/j.1574-6941.2011.01275.x 22150211

[B29] Lundquist E. J. Scow K. M. Jackson L. E. Uesugi S. L. Johnson C. R. (1999). Rapid response of soil microbial communities from conventional, low input, and organic farming systems to a wet/dry cycle. Soil Biol. Biochem. 31, 1661–1675

[B30] McCaig A. E. Embley T. M. Prosser J. I. (1994). Molecular analysis of enrichment cultures of marine ammonia oxidizers. FEMS Microbiol. Lett. 120, 363–368 8076810 10.1111/j.1574-6968.1994.tb07059.x

[B31] McCarty G. W. Bremner J. M. (1986). Inhibition of nitrification in soil by acetylenic compounds. Soil Sci. Soc. Am. J. 50, 1198–1201

[B32] Nelson D. W. (1983). Determination of ammonium in KCl extracts by the salicylate method. Commun. Soil Sci. Plant Anal. 14, 1051–1062

[B33] Nicol G. W. Leininger S. Schleper C. Prosser J. I. (2008). The influence of soil pH on the diversity, abundance and transcriptional activity of ammonia oxidizing archaea and bacteria. Environ. Microbiol. 10, 2966–2978 10.1111/j.1462-2920.2008.01701.x 18707610

[B34] Offre P. Prosser J. I. Nicol G. W. (2009). Growth of ammonia-oxidizing archaea in soil microcosms is inhibited by acetylene. FEMS Microbiol. Ecol. 70, 99–108 10.1111/j.1574-6941.2009.00725.x 19656195

[B35] Okano Y. Hristova K. R. Leutenegger C. M. Jackson L. E. Denison R. F. Gebreyesus B. Lebauer D. Scow K. M. (2004). Application of real-time PCR to study effects of ammonium on population size of ammonia-oxidizing bacteria in soil. Appl. Environ. Microbiol. 70, 1008–1016 10.1128/AEM.70.2.1008-1016.2004 14766583 PMC348910

[B36] Orlando J. Carú M. Pommerenke B. Braker G. (2012). Diversity and activity of denitrifiers of Chilean arid soil ecosystems. Front. Microbio. 3:101 10.3389/fmicb.2012.00101 22493591 PMC3319911

[B37] Pester M. Schleper C. Wagner M. (2011). The Thaumarchaeota: an emerging view of their phylogeny and ecophysiology. Curr. Opin. Microbiol. 14, 300–306 10.1016/j.mib.2011.04.007 21546306 PMC3126993

[B38] Prosser J. I. Nicol G. W. (2008). Relative contributions of archaea and bacteria to aerobic ammonia oxidation in the environment. Environ. Microbiol. 10, 2931–2941 10.1111/j.1462-2920.2008.01775.x 18973620

[B39] Purkhold U. Pommerening-Röser A. Juretschko S. Schmid M. C. Koops H. P. Wagner M. (2000). Phylogeny of all recognized species of ammonia oxidizers based on comparative 16S rRNA and *amoA* sequence analysis: implications for molecular diversity surveys. Appl. Environ. Microbiol. 66, 5368–5382 10.1128/AEM.66.12.5368-5382.2000 11097916 PMC92470

[B40] Rotthauwe J. H. Witzel K. P. Liesack W. (1997). The ammonia monooxygenase structural gene *amoA* as a functional marker: molecular fine-scale analysis of natural ammonia-oxidizing populations. Appl. Environ. Microbiol. 63, 4704–4712 9406389 10.1128/aem.63.12.4704-4712.1997 PMC168793

[B41] Schaeffer S. M. Evans R. D. (2005). Pulse additions of soil carbon and nitrogen affect soil nitrogen dynamics in an arid Colorado Plateau shrubland. Oecologia 145, 425–433 10.1007/s00442-005-0140-2 16001224

[B42] Schimel D. S. Braswell B. H. Parton W. J. (1997). Equilibration of terrestrial water, nitrogen and carbon cycles. Proc. Natl. Acad. Sci. U.S.A. 94, 8280–8283 11607734 10.1073/pnas.94.16.8280 PMC33720

[B43] Schimel J. Balser T. C. Wallenstein M. (2007). Microbial stress-response physiology and its implications for ecosystem function. Ecology 88, 1386–1394 17601131 10.1890/06-0219

[B44] Schleper C. (2010). Ammonia oxidation: different niches for bacteria and archaea? ISME J. 4, 1092–1094 10.1038/ismej.2010.111 20631805

[B45] Schwinning S. Sala O. E. (2004). Hierarchical organization of resource pulse responses in arid and semiarid ecosystems. Oecologia 141, 211–220 10.1007/s00442-004-1520-8 15034778

[B46] Shen J. P. Zhang L. M. Zhu Y. G. Zhang J. B. He J. Z. (2008). Abundance and composition of ammonia-oxidizing bacteria and ammonia-oxidizing archaea communities of an alkaline sandy loam. Environ. Microbiol. 10, 1601–1611 10.1111/j.1462-2920.2008.01578.x 18336563

[B47] Silvester W. B. Balboa O. Y. Martinez J. A. (1985). Nodulation and nitrogen fixation in members of the Rhamnaceae (*Colletia*, *Retanilla*, *Talguenea*, *Trevoa*) growing in the Chilean matorral. Symbiosis 1, 29–38

[B48] Singh J. S. Kashyap A. (2006). Dynamics of viable nitrifier community N-mineralization and nitrification in seasonally dry tropical forests and savanna. Microbiol. Res. 161, 169–179 10.1016/j.micres.2005.07.009 16427522

[B49] Stark J. M. Firestone M. K. (1995). Mechanisms for soil moisture effects on activity of nitrifying bacteria. Appl. Environ. Microbiol. 61, 218–221 16534906 10.1128/aem.61.1.218-221.1995 PMC1388328

[B50] Stopnišek N. Rangin C. G. Hoffëler S. Nicol G. W. Mandić-Mulec I. Prosser J. I. (2010). Thaumarchaeal ammonia oxidation in an acidic forest peat soil is not influenced by ammonium amendment. Appl. Environ. Microbiol. 76, 7626–7634 10.1128/AEM.00595-10 20889787 PMC2976176

[B51] Tourna M. Freitag T. E. Nicol G. W. Prosser J. I. (2008). Growth, activity and temperature responses of ammonia-oxidizing archaea and bacteria in soil microcosms. Environ. Microbiol. 10, 1357–1364 10.1111/j.1462-2920.2007.01563.x 18325029

[B52] Treusch A. H. Leininger S. Schleper C. Kietzin A. Klenk H. P. Schuster S. C. (2005). Novel genes for nitrite reductase and Amo-related proteins indicate a role of uncultivated mesophilic crenarchaeota in nitrogen cycling. Environ. Microbiol. 7, 1985–1995 10.1111/j.1462-2920.2005.00906.x 16309395

[B53] Tuba H. Boon N. Wittebolle L. Marzorati M. (2009). Environmental factors shaping the ecological niches of ammonia-oxidizing archaea. FEMS Microbiol. Rev. 33, 855–869 10.1111/j.1574-6976.2009.00179.x 19453522

[B54] Ward B. B. O'Mullan G. D. (2002). Worldwide distribution of *Nitrosococcus oceani*, a marine ammonia-oxidizing gamma-proteobacterium, detected by PCR and sequencing of 16S rRNA and *amoA* genes. Appl. Environ. Microbiol. 68, 4153–4157 10.1128/AEM.68.8.4153-4157.2002 12147525 PMC124008

[B55] Webster G. Embley T. M. Prosser J. I. (2002). Grassland management regimens reduce small scale heterogeneity and species diversity of α-protobacteria ammonia oxidizer populations. Appl. Environ. Microbiol. 68, 20–30 10.1128/AEM.68.1.20-30.2002 11772604 PMC126539

[B56] Weisburg W. G. Barns S. M. Pelletier D. A. Lane D. J. (1991). 16S ribosomal DNA amplification for phylogenetic study. J. Bacteriol. 173, 697–703 1987160 10.1128/jb.173.2.697-703.1991 PMC207061

[B57] Whitford W. G. (1992). Biogeochemical consequences of desertification. ACS Symp. Ser. 483, 352–359

[B58] Yang J. E. Skogley E. O. Schaff B. E. Kim J. J. (1998). Simple spectrophotometric determination of nitrate in water, resin, and soil extracts. Soil Sci. Soc. Am. J. 62, 1108–1115

[B59] Yeager C. M. Northup D. E. Grow C. C. Barns S. M. Kuske C. R. (2005). Changes in nitrogen-fixing and ammonia-oxidizing bacterial communities in soil of a mixed conifer forest after wildfire. Appl. Environ. Microbiol. 71, 2713–2722 10.1128/AEM.71.5.2713-2722.2005 15870363 PMC1087562

[B60] Zhang L. M. Offre P. R. He J. Z. Verhamme D. T. Nicol G. W. Prosser J. I. (2010). Autotrophic ammonia oxidation by soil Thaumarchaea. Proc. Natl. Acad. Sci. U.S.A. 107, 17240–17245 10.1073/pnas.1004947107 20855593 PMC2951460

